# A proposed model to conduct process and outcome evaluations and implementation research of child health programs in Africa using integrated community case management as an example

**DOI:** 10.7189/jogh.04.020409

**Published:** 2014-12

**Authors:** Theresa Diaz, Tanya Guenther, Nicholas P Oliphant, Maria Muñiz, the iCCM Symposium impact outcome evaluation thematic group

**Affiliations:** 1 UNICEF, Programme Division, Health, New York, NY, USA; 2 Save the Children, Washington DC, USA; * Contributors from the iCCM Symposium impact outcome evaluation thematic group are listed at the end of the article.

## Abstract

**Aim:**

To use a newly devised set of criteria to review the study design and scope of collection of process, outcomes and contextual data for evaluations and implementation research of integrated community case management (iCCM) in Sub–Saharan African.

**Methods:**

We examined 24 program evaluations and implementation research studies of iCCM in sub–Saharan Africa conducted in the last 5 years (2008–2013), assessed the design used and categorized them according to whether or not they collected sufficient information to conduct process and outcome evaluations.

**Results:**

Five of the 24 studies used a stepped wedge design and two were randomized control trials. The remaining 17 were quasi–experimental of which 10 had comparison areas; however, not all comparison areas had a pre and post household survey. With regard to process data, 22 of the studies collected sufficient information to report on implementation strength, and all, except one, could report on program implementation. Most common missing data elements were health facility treatments, service costs, and qualitative data to assess demand. For the measurement of program outcomes, 7 of the 24 studies had a year or less of implementation at scale before the endline survey, 6 of the household surveys did not collect point of service, 10 did not collect timeliness (care seeking within 24 hours of symptoms) and 12 did not have socioeconomic (SES) information. Among the 16 studies with comparison areas, only 5 randomly selected comparison areas, while 10 had appropriate comparison areas.

**Conclusions:**

Several evaluations were done too soon after implementation, lacked information on health facility treatments, costs, demand, timeliness or SES and/or did not have a counterfactual. We propose several study designs and minimal data elements to be collected to provide sufficient information to assess whether iCCM increased timely coverage of treatment for the neediest children in a cost–efficient manner.

Evaluation and implementation research of the delivery of maternal, newborn and child health interventions that focus on impact, specifically those assessing changes in morbidity or mortality, have been deemed critical to determine the effectiveness of programs being implemented [1]. However, unlike an efficacy trial, in reality evaluators and researchers cannot control programs implemented by governments, international or bilateral agencies, and private voluntary organizations, nor can they control contextual factors that affect the program [2,3]. In sub–Saharan African countries and many other developing countries, multiple organizations support different programs and may work in different areas of the same country, implement their programs differently and may implement in areas that were designated to be the control arm of an evaluation without the evaluator being aware. In addition, achieving full implementation at scale can take much longer than anticipated and other contextual factors not under the control of the evaluator, such as national stock out of medications, can negatively impact the programs. Without documenting these factors and other details of implementation, the impact studies are limited in their ability to explain why programs do or do not produce expected impact.

Therefore measuring impact should not be the only focus of evaluations or implementation research of maternal, newborn or child health programs that are delivering proven interventions, but rather it should be supplemented with measures of process and outcomes coupled with contextual information to better understand if and how (or if not and why not) programs are providing the interventions to those in need. Process evaluations measure the internal dynamics of implementing organizations, their policy instruments, their service delivery mechanisms, and their management practices [4]. Specifically, they determine what is done by the program, and for whom these services are provided [5]. Outcome evaluations measure the likely or achieved short– and medium–term effects of an intervention’s outputs [4] such as behavior change (eg, what proportion of those who might need the service(s) sought care) and coverage (eg, what proportion of those who might need the service received them). Contextual factors can include factors that may impact implementation such as the functioning of the overall health system or factors that may affect the impact of the intervention such as the socioeconomic factors and status or underlying health status of the population [3].

The problem with evaluations that focus on impact, without examining process, outcomes and context can be highlighted with one particular child health program, integrated community case management (iCCM). Around 2008–9, funding levels to support iCCM expansion in sub–Saharan Africa increased substantially. However, a recent review of published evaluations in Africa concluded there is no evidence of mortality impact of community–based pneumonia treatment [6]. This conclusion does not make sense given that earlier impact evaluations of iCCM, mostly in Asia, have shown that using community health workers (CHWs) to deliver treatments can reduce pneumonia specific and overall mortality in children and the fact that that provision of antibiotics for pneumonia is effective at reducing mortality in children [7–9]. In actuality, many of the reviewed studies in sub–Saharan Africa focused on specific aspects of the program, and not the entire process and outcome. Most of the included studies concentrated on measuring CHWs adherence to established guidelines [10]. Complete evaluations that included process, outcomes and context were missing.

Several researchers have proposed evaluation methods that may be able to take into account process, outcome and context. Realist evaluations, which is a form of theory driven evaluation that is context–specific, represent one example [11]. In this type of evaluation, interventions work (or not) because people make particular decisions in response to what is provided by the intervention (or not) in a particular context. Their response to the resources, or opportunities provided by the intervention is what causes the outcomes. Measuring contextual factors matters because these factors influence their response; measuring process is important to understand how and why decisions were made. Another more recent suggestion for evaluations of large–scale programs and initiatives in middle– and low–income countries is a national platform approach [12]. This evaluation approach uses a geographic unit (usually district) as the unit of analysis. Relevant information from existing databases would be integrated in a continuous manner into one data repository. New information about program implementation by different agencies (government, bilaterals, multilaterals, non–governmental organizations [NGOs]) would also be included in the database. It would also include contextual information. Although the focus would be on using existing data, additional data on program management and data quality may need to be collected and added. Comparisons of different geographic units could be done either based on a score or dose–response analyses of program implementation strength and coverage. For both these types of evaluations, additional data to assess process, outcome and contextual factors need to be collected.

With the increase in funding for iCCM there has been a great demand from donors for evaluations and implementation research largely focused on measuring the impact, specifically mortality, of these programs. Despite the pressure to focus on mortality impact many evaluators and implementers recognized the importance of documenting and measuring implementation, and therefore examined process, outcomes and context, but used different methods and data elements. Our objective was to develop and apply a newly devised set of criteria to review the study design and scope of collection of process, outcomes and contextual data for evaluations and implementation research of iCCM) in Sub–Saharan African and to propose an evaluation study design(s) based on gaps identified. We examined 24 evaluations and/or implementation research studies of iCCM that were completed in the last 5 years (2008–2013).

## METHODS

### Identification of evaluations

We searched for completed evaluations or implementation research studies of iCCM with endpoints that included outcomes and/or impact measures, conducted between 2008 and 2013. For purposes of this assessment implementation research was defined as studies that went beyond measuring outcomes or impact and also examined what was happening with implementation within existing health systems to determine what worked or did not work and why. The CHWs must have treated at least 2 of the three conditions (malaria, pneumonia and diarrhea) and the evaluation must have included a measure of coverage or mortality. We contacted the key international NGOs supporting iCCM implementation, universities known to be involved in evaluating iCCM, UNICEF, and WHO to create a list of all the iCCM evaluations undertaken in Africa conducted in the last 5 years (2008–2013). We identified 23 evaluations that met these criteria. We then did a PubMed search for articles from 2008 to 2013 using key words “community case management, Africa, children” and also “integrated community case management, Africa, Children” and found a total of 213 articles of which only 14 were outcome or impact evaluations, or implementation research. All except 3 had already been identified by contacting researchers. The other three articles [13–15] were from one study, and were included as well for a total of 24 evaluations. We asked the principal investigator of each evaluation to complete a standardized Excel spreadsheet on the characteristics of their evaluation and the programs being evaluated. We asked each researcher to describe the study design, whether pre and post household surveys were conducted, if yes, what was the first and second level of selection, how were households selected, what were the sample sizes, who conducted the interviews, who designed the study and who did the training. We also asked whether or not there were comparison areas and how they were selected. We also asked the population size of the iCCM program area.

### Development of evaluation questions and framework

Although some studies assessed specific aspects of community–based treatment, we needed to first frame our review of research and evaluation designs by developing an overall evaluation question about iCCM. This question was:

1. *What is the contribution of iCCM to reduction of childhood morbidity and mortality in African countries?*

We then determined the set of questions that needed to be answered to address this overall evaluation question. These questions were:

a. Did iCCM accelerate coverage of appropriate and timely treatment for pneumonia, malaria and diarrhea (or at least two of these conditions if only two conditions were being treated at the community level) in children? (i) If yes, how?, (ii) If no, why not?

b. Did iCCM decrease the inequities in treatment coverage for pneumonia, malaria and diarrhea in children? (i) If yes, how?, (ii) If no, why not?

**Figure 1:**
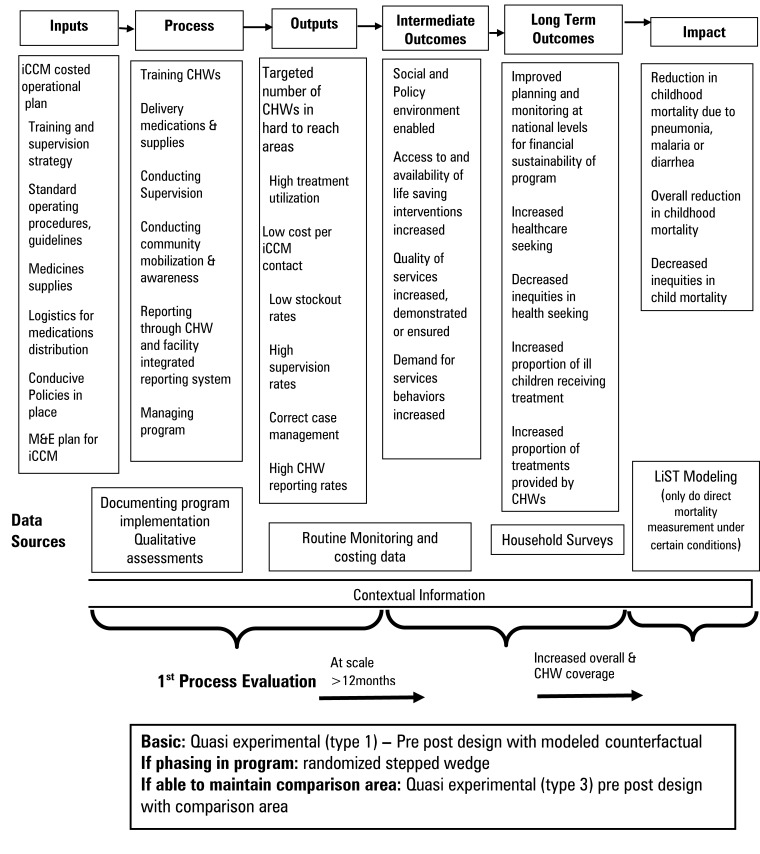
Evaluation framework for integrated community case management program. iCCM – Integrated Community Case Management, CHWs – Community Health Workers, M&E – Monitoring and Evaluation, LiST – Lives Saved Tool.

We then developed a monitoring and evaluation framework to answer the evaluation questions we proposed to allow us to categorize the study designs under review. This framework is based on the theory of change that framed the overall iCCM evidence symposium [16] and was also adapted from previous frameworks for evaluation of child health and community–based care programs (**Figure 1**) [17–19]. The data sources needed to assess different components of the model were listed and how these data components are related to process, outcome and impact evaluation are shown. We listed the sequence in which evaluations should be done, starting with process evaluation to assess if program implementation was at scale and of adequate strength, then outcome evaluation to determine if there were increases in coverage and if CHWs contributed to this increase, and finally impact evaluation. Finally, we listed the study design options that may be considered to answer the evaluation questions.

### Analysis of evaluations

We assessed if the evaluation design and data collected, amongst the sample of evaluations identified, were consistent with our framework. Specifically we determined if they had sufficient data to conduct the process and outcome evaluations, if comparison areas were adequate and if results had been disseminated. Given that we did not have raw data and the paucity of dissemination, we did not assess the quality of the data or the quality of the analysis conducted.

First we classified evaluations based on their design, study area and time frame. Cluster randomized stepped wedge trials were those studies in which a sequential roll–out of an iCCM program was implemented in randomly selected clusters over a number of time periods. By the end of the study, all clusters had received iCCM, although the order in which the cluster received iCCM was determined at random. A cluster randomized control trial was one in which entire unit or clusters of health care providers (eg, CHWs) rather than independent individuals were randomly allocated to intervention and comparison groups. (Pre–test was defined as having collected coverage data at baseline. Post–test was defined as having collected coverage data at the end of the evaluation period.). Quasi–experimental trials were those studies that had pre and post tests. Some of these studies had comparison areas.

After describing the programs, we reviewed the reported information based on a set of criteria for process evaluation and outcome evaluation. In addition, for those evaluations with comparison areas we also assessed if those comparison areas were adequate. Finally, we described the dissemination activities of each evaluation, to determine if final analysis, reports are completed and available to the public and what is planned for the future.

The criteria we used to assess process evaluation were based on whether there was data collected to assess the strength of program implementation, treatment rates, costs, context, demand, and program implementation. Because we used iCCM as a model, we based our data elements on those data needed to construct indicators in each of these areas as defined in the CCM indicator guide [17]. For implementation strength this included: data on number of CHWs per under 5 population; data on supervision (whether from routine reports or cross-sectional survey); data on stockouts (whether from routine reports or cross–sectional surveys). For treatment rates, we determined if there was routine reporting data from CHWs on number of treatments for pneumonia, malaria and diarrhea, rates of reporting from CHWs and routine reporting on treatments for these diseases from facilities. For the remaining data elements, we assessed whether costing data was available; whether there was qualitative data from caregivers on health seeking or from health staff, CHWs, health managers on impressions of the program; whether program management was documented (specifically whether training process, supervision procedures, supply logistics and distribution were described); and contextual data (specifically whether there was information collected on outside influences that can affect implementation such as natural disasters, fuel shortages, strikes, national level stockouts of medications, other programs in the same areas or factors that can affect impact such as socioeconomic status, immunization rates and other health status information).

A recent review of Demographic and Health Surveys (DHS) and Multiple Indicator Cluster Surveys (MICS) assessed the extent to which large–scale national household surveys can serve as sources of baseline data for evaluating trends in community–based treatment coverage for childhood illnesses [20]. The authors suggested that the place and provider of treatment needed to be collected. This point of service information as well as additional criteria to ensure the program had been in place long enough to see outcomes; coverage data reflective of the program area and including timeliness of treatments (health care seeking within 24 hours); and socioeconomic status, are all important. Therefore, we assessed the outcome data elements based on whether there was: 1) greater than one year of implementation at scale (ie, 80% of the target CHWs were trained and deployed for greater than a year) before the endline survey was completed; 2) a baseline household survey of caregivers on health seeking and treatment; 3) information on where/from whom the treatment was received, timeliness of treatment and wealth captured at baseline; 4) an endline household survey of caregivers on health seeking and treatment; 5) information on where/from whom the treatment was received and timeliness of treatment and wealth captured in the endline survey; and 6) baseline and endline surveys conducted in areas representative of where the intervention took place (eg, in the district or village where iCCM was taking place rather than extrapolating from a regional or national survey).

We assessed comparison area adequacy based on the following criteria: 1) if the comparison area was appropriate for the research question; 2) if there were the at least the same number of comparison areas as intervention areas, 3) how the comparison area was selected, and 4) if there were no issues with comparability reported by the researchers.

## RESULTS

Of the 24 evaluations, 5 used a stepped wedge randomized controlled design although there were slight variations in randomization procedures (**Table 1**). Two of these five studies had three arms comparing adding pneumonia treatment to malaria treatment. One of these five evaluations used a randomized cluster design with stratification by zone, one was a mixture of stepped–wedge and quasi–experimental and one was semi–randomized. Two studies were randomized control trials that were not stepped–wedge. Seventeen studies were quasi–experimental, 10 of which had comparison areas. Five studies conducted only a post coverage survey in the comparison area. Seven studies had pre and post survey data on coverage but no comparison areas.

**Table 1:** Study descriptions (see Online Supplementary Document(Online Supplementary Document))

Country (Study organization)	Design
**1. Burkina Faso** (Special Programme for Research and Training in Tropical Diseases, WHO, Groupe de Recherche Action en Santé)	Cluster–randomized stepped wedge (three arms).
**2. Cameroon** (Population Services International)	Quasi–experimental pre–test/post–test
**3. Ethiopia Oromia** (John Hopkins University)	Cluster–randomized step–wedge with stratification by zone
**4. Ghana** (Special Programme for Research and Training in Tropical Diseases, WHO, School of Public Health, University of Ghana)	Cluster–randomized stepped wedge
**5. Ghana** (South Africa Medical Research Council) on behalf of UNICEF)	Pre–test/post–test no comparison area
**6. Malawi** (Save the Children)	Quasi–experimental step–wedge with comparison at midline but all areas with intervention at endline
**7. Malawi** (South Africa Medical Research Council on behalf of UNICEF)	Pre–test/post–test no comparison area
**8. Mozambique** (Save the Children)	Quasi–experimental post–test only with comparison area
**9. Niger** (South Africa Medical Research Council on behalf of UNICEF)	Pre–test/post–test no comparison area
**10. Rwanda** (International Rescue Committee)	Pre–test/post–test no comparison area
**11. Sierra Leone** (International Rescue Committee)	Semi–randomized stepped wedge trial design.
**12. Sierra Leone** (UNICEF)	Quasi–experimental pre–test/post–test
**13. South Sudan** (International Rescue Committee)	Quasi–experimental pre–posttest intervention area, comparison area post–test only
**14. South Sudan** (Malarial Consortium)	Quasi–experimental pre–posttest intervention area, comparison area post–test only
**15. South Sudan** (Save the Children)	Quasi–experimental pre–posttest intervention area, comparison area post–test only
**16. Uganda Central** (Malaria Consortium and UNICEF)	Quasi–experimental pre–test/post–test
**17. Uganda East** (Special Programme for Research and Training in Tropical Diseases, WHO, Makerere University)	Cluster–randomized trial
**18. Uganda West** (Malaria Consortium)	Quasi–experimental pre–test/post–test
**19. Zambia** (Malaria Consortium)	Quasi–experimental, comparison at post test
**20. Ethiopia** (South Africa Medical Research Council on behalf of UNICEF)	Pre–test/post–test no comparison area
**21. Mali** (South Africa Medical Research Council on behalf of UNICEF)	Pre–test/post–test no comparison area
**22. Mozambique** (South Africa Medical Research Council on behalf of UNICEF)	Pre–test/post–test no comparison area
**23. Mozambique** (Malaria Consortium)	Pre–test/post–test no comparison area
**24. Zambia** (Boston University)	Cluster–randomized controlled trial

**Table 2** shows which of the data elements needed to conduct a process evaluation were collected for each evaluation. All, except one, of the evaluations were able to report on program implementation and 22 on all the implementation strength indicators. The most common missing data elements were: CHW reporting rates (12 missing), health facilities treatments (13 missing), costing (12 missing) and qualitative data from caregivers (14 missing). With regard to contextual factors data, 18 studies had data on factors that affect impact, whereas fewer (10) studies had data on factors that affect implementation.

**Table 2:** Process evaluation data criteria: Whether or not data element listed was collected as part of the study

	Implementation strength			Reporting data			Cost, demand, management and contextual data*			
**Study number**	**Number of CHWs and population covered**	**Supervision rates**	**Stock–outs**	**CHW reporting rates**	**Number of CHW treatments**	**Facility based treatments**	Cost data*	**Context – implementation† or impact‡ or both**	**Qualitative data caregiver§ or health provider¶**	**Program management#**
**1**	Yes	Yes	Yes	Yes	Yes	Yes	No	Impact	No	Yes
**2**	Yes	Yes	Yes	No	Yes	No	No	Impact	Both	Yes
**3**	Yes	Yes	Yes	No	No	No	No	Both	Both	Yes
**4**	Yes	No	Yes	No	Yes	Yes	Yes	Impact	Both	Yes
**5**	Yes	Yes	Yes	No	Yes	No	Yes	Impact	Health provider	Yes
**6**	Yes	Yes	Yes	Yes	Yes	Yes	No	No	No	Yes
**7**	Yes	Yes	Yes	No	Yes	No	Yes	Both	Health provider	Yes
**8**	Yes	Yes	Yes	Yes	Yes	Yes	No	No	No	Yes
**9**	Yes	Yes	Yes	No	Yes	Yes	Yes	Both	Health provider	Yes
**10**	Yes	Yes	Yes	Yes	Yes	No	No	Both	Health provider	Yes
**11**	Yes	Yes	Yes	Yes	Yes	Yes	No	Impact	Health provider	Yes
**12**	Yes	Yes	Yes	No	Yes	Yes	No	Both	Both	Yes
**13**	Yes	Yes	Yes	Yes	Yes	No	No	Impact	No	Yes
**14**	Yes	Yes	Yes	Yes	Yes	No	No	No	No	Yes
**15**	Yes	Yes	Yes	Yes	Yes	No	No	No	No	Yes
**16**	Yes	Yes	Yes	Yes	Yes	Yes	No	Both	Both	Yes
**17**	Yes	No	Yes	No	Yes	No**	Yes	Impact	Both	Yes
**18**	Yes	Yes	Yes	Yes	Yes	Yes	Yes	Impact	Both	Yes
**19**	Yes	Yes	Yes	Yes	Yes	No	Yes	No	Both	Yes
**20**	Yes	Yes	Yes	No	Yes	No	Yes	Both	Health provider	Yes
**21**	Yes	Yes	Yes	No	Yes	Yes	Yes	Both	Health provider	Yes
**22**	Yes	Yes	Yes	No	Yes	No	Yes	Both	Health provider	Yes
**23**	Yes	Yes	Yes	No	Yes	No	Yes	Both	Both	Yes
**24**	Yes	Yes	Yes	Yes	Yes	Yes	Yes	No	Both	No

*Cost data were collected separately from the evaluations by Management Sciences for Health in Cameroon, Sierra Leone, South Sudan, and Zambia for purposes of comparison with the evaluation findings. These comparisons have not yet, however, been made.

†Collected information on outside influences that could affect implementation such as other programs in the area, national stock outs of medications, changes in policies.

‡Collected information on factors that could affect impact such as socioeconomic status or health status of population.

§Focus groups or key informant interviews from caregivers or community on health seeking practices and/or barriers.

¶Focus groups or key informant interviews from health providers, implementers, health managers on aspects of program implementation.

#Able to report on training procedures, supervision procedures, supply/medicine logistics and distribution procedures.

**Did report on malaria incidence in facilities but not on treatments.

We found that 7 studies were conducted of programs that were implementing at scale for a year or less (**Table 3**). Six studies had no information on point of service, 10 did not collect data on timeliness and 12 did not have wealth information either in the baseline or endline survey. Of the 16 studies with comparison areas, 6 had no baseline survey in the comparison area (note that three studies in Southern Sudan used the same comparison area). Although all were conducted in the intervention areas, some relied on pooling regional data or using the rural component of larger surveys. Others just sampled from target areas, not necessarily ensuring that the selected clusters were in fact exposed to iCCM. Of those who conducted household surveys for the purposes of the iCCM evaluation only, most were two–stage cluster surveys but some used Lot Quality Assurance Sampling.

**Table 3:** Outcome (coverage) data: Whether or not data element listed was collected as part of the household survey data

		Pre					Post				
**Study number**	Months of implementation at scale* by endline	**household survey**	**Includes point of service**	**Includes timeliness**	**Wealth data (assets)**	**Conducted in Intervention (and/or control area)**	**Household survey**	**Includes point of service**	**Includes timeliness**	**Wealth data (assets)**	**Conducted intervention area (and or control area)**
**1**	24	Yes†	No	No	No	Yes	Yes†	No	No	No	Yes
**2**	36	Yes‡	Yes§	No	Yes	Yes	Yes‡	Yes§^4^	No	Yes	Yes
**3**	24	Yes‡	Yes	No	No	Yes	Yes‡	Yes	No	Yes	Yes
**4**	24	Yes†	No	No	No	Yes	Yes†	No	No	No	Yes
**5**	12	Yes¶	Yes	Yes**	No	Yes	Yes#	Yes	Yes	No	Yes
**6**	17	Yes‡	Yes	Yes**	Yes	Yes	Yes‡	Yes	Yes†	Yes	Yes
**7**	11	Yes¶	No	No	No	Yes	Yes#	Yes	Yes	No	Yes
**8**	28	Yes‡	No	No	No	No in control area	Yes‡	Yes	Yes**	No	Yes
**9**	35	Yes¶	Yes	Yes	Yes	Yes	Yes¶	Yes	Yes	Yes	Yes
**10**	42	Yes	No	Yes**	No	Yes	Yes	No	Yes**	No	Yes
**11**	24	Yes‡	No	No	No	Yes	Yes‡	No	No	No	Yes
**12**	17	Yes‡	Yes	No	Yes	Yes	Yes‡	Yes	No	Yes	Yes
**13**	>72	Yes‡	Yes	Yes	No	No in control area	Yes‡	Yes	Yes	No	Yes
**14**	12	Yes‡	Yes	Yes	No	No in control area	Yes‡	Yes	Yes	No	Yes
**15**	21	Yes‡	Yes	Yes	No	No in control area	Yes‡	Yes	Yes	No	Yes
**16**	22	Yes‡	Yes	Yes	Yes	Yes	Yes‡	Yes	Yes	Yes	Yes
**17**	24	Yes†	No	No	No	Yes	Yes†	No	No	No	Yes
**18**	22	Yes‡	Yes††	Yes	Yes	Yes	Yes‡	Yes††	Yes	Yes	Yes
**19**	24	Yes‡	Yes††	Yes	Yes	No in control area	Yes‡	Yes††	Yes	Yes	Yes
**20**	12	Yes¶	Yes	Yes	Yes	Yes	Yes¶	Yes	No	Yes	No
**21**	<12	Yes‡‡	Yes	Yes	Yes	Yes	Yes#	Yes	Yes	Yes	Yes
**22**	<12	Yes‡^3^	Yes	No	Yes	Yes	Yes¶^5^	Yes	No	Yes	Yes
**23**	24	Yes‡^3^	No	Yes	Yes	Yes	Yes‡^3^	No	Yes	Yes	Yes
**24**	12	Yes‡^3^	Yes	Yes	Yes	Yes	Yes‡^3^	Yes	Yes	Yes	Yes

*80% of targeted Community Health Workers deployed and active.

†Demographic Sentinel Surveillance site collected mortality data biannually.

‡Household cluster survey conducted for the purposes of the evaluation.

§For Oral Rehydration Solution only.

¶Used existing national level household surveys such as Multiple Indicator Survey or Demographic and Health Survey. If program was not national restricted analysis to areas where intervention were taking place either at the district or regional area based on what was feasible from the original sampling design.

#Use Lot Quality Assurance Survey.

**For Treatment of Fever and Pneumonia.

††Community Health Worker only.

‡‡Demographic Household Survey was aggregated into 5 regions were program was operating, but included urban areas (exclusive of Bamako).

Among the 16 studies with comparison areas, 6 had comparison areas in which CHWs were providing treatments for at least one of the three illnesses, usually malaria, but this was appropriate for the study design (**Table 4**). Only 5 selected comparison areas randomly. Of the 16 studies with comparison areas, 11 reported that the comparison area was similar to the intervention area; however, 10 were appropriate comparison for the question being asked. The one study that did not have an appropriate comparison area was evaluating the outcome and impact of iCCM but had iCCM taking place in the comparison area. There were some differences in the number of intervention and comparison areas, they were not always evenly matched although all investigators claimed their studies were powered to test the main outcome (coverage).

**Table 4:** Description of studies with comparison areas

Study number	Comparison area without any iCCM	Number of comparison areas in relation to intervention area	Comparison area selection	Comparison area similar to intervention area at baseline	Appropriate comparison areas*
**1**	No, CHWs treating malaria	19 control clusters + 38 intervention clusters of 2 districts	Random	Yes	Yes
**2**	Yes	2 intervention + 1 control of 20 districts	Purposive sampling	Yes	Yes
**3**	No, CHWs treated diarrhea and malaria	16 intervention + 15 control woredas	Restricted randomized selection	Yes	Yes
**4**	Yes	1 district with 114 clusters of which 37 randomized to one intervention arm, 39 to another intervention arm and 38 to the control	Random	No	Yes
**6**	No at endline yes at midline	70 clusters of 20 households (1400 households total); evenly divided between phase 1 and phase 2 areas	Areas that were 8+ km from a health facility as identified by district health officials and who did not have an CHWs trained in iCCM	Yes	Yes
**8**	No	3 intervention + 1 comparison district	Selected because it is a large district adjacent to one of the intervention districts with few CHWs	Yes	Yes
**11**	Yes	4 of 12 districts	Semi–randomized	Yes	Yes
**12**	Yes	2 implementing + 2 control districts	Similarity to intervention areas on a several of key health indicators	No	No
**13**	Yes	1 intervention county + 1 control county	Similarity to intervention areas	No	No
**14**	Yes	1 intervention county + 1 control county	Similarity to intervention areas	No	No
**15**	Yes	2 intervention counties + 1 control county	Similarity to intervention areas	No	No
**16**	Yes	3 intervention + 3 control districts	Used comparison area that was already selected for Uganda west study	No	No
**17**	Yes	2 districts with villages randomized into control and intervention areas	Randomized	Yes	Yes
**18**	Yes	8 intervention and 3 control districts	Districts where iCCM has not been implemented but with similar demographic profile to intervention districts	Yes	Yes
**19**	No, districts where iCCM had been implementing for up to 8 mo	4 intervention and 3 control districts (phased–in)	Districts where iCCM had been implementing for up to 8 months	Yes	No
**24**	No, CHWs treated for presumptive malaria based on fever	Yes	Yes	Yes	Yes

iCCM – integrated community case management, CHWs – community health workers

*Appropriate for research question, similar to intervention area.

Finally, regarding dissemination of findings, of the 14 evaluations that were completed (eg, all data analysis had been completed) seven evaluations were published in the peer–reviewed literature. The TDR WHO studies of Burkina Faso, Ghana and Uganda East had multiple publications based on endline evaluations [8,21–23] as did ZIMMAPS [13–15] There were also publications of the midterm evaluation in Cameroon [24] and a component of the Ethiopian JHU evaluation [25]. Although Sierra Leone UNICEF had multiple publications, those only presented baseline data [26–29]. All the remaining completed evaluations had reports but none were available to the public. The other evaluations were in the process of finalizing endline reports.

## DISCUSSION

Our review of the process, outcome and contextual data elements from 24 recent evaluations and implementation research studies of iCCM found that the most commonly missing information for process data were reporting rates of CHWs, facility treatments, costing data and qualitative data. For outcome data, many of the surveys did not collect point of service, timeliness or wealth information, which would make it difficult to fully determine the contribution of CHWs with regard to coverage and equity. In addition, several of the household surveys were not reflective of the area where the program was taking place but rather a larger geographic area (eg, regional level data as a proxy for a district within the region) and ignored the possibility that randomly selected clusters may have altogether missed the communities exposed to iCCM if CHWs were only deployed in hard to reach areas. This was true among the evaluations, mostly UNICEF supported, which at the request of governments and due to donor funding constraints, pre–existing surveys were used to save on labor, time and cost. Only recently has point of service and timeliness been included in these larger household surveys (eg, DHS and MICS) [20]. Many of the designs observed did not include comparison areas. Some type of counterfactual is necessary to understand the contribution of CHWs to outcomes and impacts. Of those that did have comparison areas, few were chosen randomly but slightly more than half of the researchers reported that the comparison area was similar to the intervention area at baseline. Additionally, the number of interventions and comparison areas was not always evenly matched although all investigators claimed their studies were powered to test the main outcome.

Our review informs the feasibility, opportunities, and constraints for design options (**Table 5**). Although realist evaluations are feasible and opportunities to conduct these exist, they are constrained by the need for additional contextual data to be collected and specific expertise to do such an analysis [11]. The evaluation platform may be an option in the future for conducting these evaluations, but has yet to be fully tested [12]. This type of evaluation will be especially useful in countries where iCCM is already taking place on a national scale. However, it is constrained by the underlying data availability within districts. If current and new evaluations, and studies collect the data elements we assessed, it could contribute to the district level databases making this type of evaluation feasible. This approach is currently being tested in four African countries (Malawi, Niger, Tanzania, and Mozambique) by Johns Hopkins University.

**Table 5:** Opportunities and constraints of possible study designs

Study Design	Opportunities	Constraints
Realist	• Can be adapted to the local setting	• Requires specific expertise • Results may be difficult to understand or explain • Requires extensive contextual information • No method to quantitatively/statistically compare to a control area
Evaluation platform	• Improvements in district level data collection increasing • Could be used for multiple programs	• Requires availability of high quality data from districts • Not yet tested
Stepped–wedge design	• When program is first being scaled up	• Need to allow for longer start–up periods • Randomization may not be allowable
Quasi–experimental (1) pretest–posttest designs without control groups	• Easier to implement than designs with control areas • Less costly than most designs	• No control area (although can consider modeling counterfactual)
Quasi–experimental (2) pretest–posttest design in the intervention area with control groups at end line	• Can select a control area in which you know the program did not exist • Less costly than having both baseline and end line data	• Assumes control area was similar at baseline • May have little information of activities in control area during intervention implementation period
Quasi–experimental (3) pretest–posttest design with control groups where both the intervention and control areas had baseline and endline surveys.	• usually acceptable to government • able to document activities in control area during implementation period	• Cannot guarantee lack of contamination of control area • Control area may not be well matched to intervention area
Randomized cluster control trial (not step wedged)	• Best in controlled environments such as DSS sites • If government agreeable to randomization at start of program	• Not feasible in most settings • May not be generalizable • Randomization may not be allowable • Resource intensive

DSS – demographic sentinel surveillance

The stepped wedge designs appeared promising. A systematic review of this design suggested that it can be used when interventions are likely to do more good than harm, when interventions are being implemented in a new setting, where evidence for their effectiveness in the original setting is available and for cost–effectiveness analyses of interventions that have already been shown to be effective [30]. With regard to opportunity, the evaluation of iCCM appears to be the ideal candidate for this design when a country is first scaling up the program. However, with regard to feasibility and constraints there are some issues, the stepped wedge design requires a longer trial duration than other designs, especially to allow for evaluating programs at scale. Additionally, there may not be an opportunity to randomize areas, and the design requires assistance from statisticians and researchers who have experience with this type of study design [30].

We identified three types of quasi–experimental study designs: 1) pretest–posttest designs without control groups, 2) pretest–posttest designs in the intervention area with control groups but the control group did not have a baseline survey and 3) pretest–posttest designs with control groups where both the intervention and control areas had baseline and endline surveys. The first design appears to be the simplest design and thus feasible; however, this design is constrained by the fact that it does not offer a counterfactual. The second design which may not always be feasible is constrained by the fact that it assumes the control area had similar rates of coverage to the intervention area at base line, but this may not be the case. Although the third design is stronger than the second design, it has constraints because the 2 groups were not selected randomly, selection bias may still exist and in fact we found some comparison areas were not similar to intervention areas [31].

Randomized control trials that are not stepped–wedge appear to be the least feasible, although there can be an opportunity to conduct such studies, especially, if the program is being implemented in a controlled environment such as a demographic sentinel surveillance site (DSS) [32]. In fact, two of our studies were randomized cluster control trials [8,13], one of which was in a DSS site in Ghana. There are constraints to conducting randomized control trials. They require specific technical expertise, and there is often an inability to completely prevent or fully measure contamination. Also because we are trying to evaluate the scale up iCCM in a real world scenario, this alternative will rarely present itself and if done in a DSS site, it will have limited generalizability.

Based on our review and the designs discussion, we propose the following options for future evaluation designs. At the least, a pre–posttest evaluation should be performed by 1) conducting a baseline household survey in the area where the intervention is taking place, which should include point of service and timeliness and socioeconomic status; 2) prospectively collect all the process and contextual data elements we assessed; 3) periodically review and analyze the process information to determine if the program is at scale (scale should be defined locally but we suggest at least 80% of the target number of CHWs are active) and of high quality (supervision rates, CHW reporting rates, and no stock–out rates of over 80%) for at least one year; 4) use qualitative data and program implementation documentation to determine barriers for reaching scale, if scale has not been reached; 5) make changes based on these findings and re–do the process evaluation once these changes are implemented; 6) estimate start–up costs and recurrent costs per iCCM service and per capita as [33]; 7) once at scale for one year (with all the previous provisos regarding implementations strength) conduct an endline coverage survey; 8) analyze this coverage survey for improvements in timeliness and decreases in inequities of health care seeking and coverage as well as cost–effectiveness; 9) only proceed further to assess mortality impact if there is a significant increase in coverage that includes a proportion of CHWs providing those treatments. If there is no increased coverage, there is no need to proceed to measure or model mortality impact as such an evaluation will only substantiate your outcome findings.

As actual measurement of mortality rates through surveys is difficult and costly, will require larger sample sizes, require additional time of implementation before being able to see impact and also specific expertise, we suggest modelling mortality impact. We recommend that mortality measurement only be done if the circumstances are present as specified in the mortality article in this journal supplement [34]. Using a model, we can also create a counterfactual by comparing the actual coverage changes to a modeled scenario as though the program did not exist. Although there are a variety of models to choose from, we suggest using the Lives Saved Tool (LiST), which is a program to project the changes in child and maternal survival in accordance with changes in coverage of different child and maternal health interventions [35]. LiST is based on a linear, mathematical model that is deterministic [36]. The relationship between changes in intervention coverage and one or more outputs (eg, cause specific mortality, lives saved) is specified in terms of the effectiveness of the intervention for reducing the probability of that outcome. Many systematic reviews have been conducted to determine the effectiveness of interventions used in the model and the program is easy to use [35]. We first suggest quantifying the estimated number of lives saved overall and separately saved by treatments provided by CHWs. Then create a counterfactual in which the CHW’s proportional contribution to increased coverage is removed but everything else is unchanged. An example of how to do this is provided in the LiST article in this supplement [37].

There are some circumstances where a comparison area is still possible. If the program is being introduced and there is agreement to do so in a staggered way and at random, we suggest considering a stepped wedge design assuming appropriate technical support can be provided. If a similar comparison area to the intervention area can be identified, and evaluators have some control over the comparison to avoid contamination during the evaluation study period, a quasi–experimental design with control groups (type 3 above) can be considered.

Regardless of which of the proposed designs are used, all process, outcome and contextual data assessed in this review must be collected and periodic analysis of these data must be done to determine how well the program is functioning to make changes as needed, as has been suggested by other frameworks for implementation research [38,39]. For any evaluation or implementation research in maternal, newborn or child health the data collected must include the appropriate numerator and denominator data to measure globally accepted standardized indicators. In the case of iCCM, these are those described in the CCM indicator handbook [17] and highlighted in the monitoring paper in this supplement [40]. Once the outcome evaluation has demonstrated a positive impact, routine monitoring should continue and process evaluation should be done periodically to assess the program. A full evaluation with pre and post household surveys should not be necessary if process data, especially examining routine reporting data, are periodically analyzed and acted on.

There are several limitations to our review. First, we did not have the raw data (eg, reporting data, household survey data, etc.) to determine the quality of the data elements. The quality is expected to be quite variable with some evaluations supported by academic institutions with experienced researchers and others mostly conducted by NGOs with variable expertise in evaluation methodologies and management. In addition, limited literacy of interviewers and CHWs and lack of qualified supervisors to manage interviewers in some countries may have compromised some reporting data and surveys. Although we did review the reports available to us, we did not assess the quality of the analysis that was done. However, we should note that all reports reviewed used a mixed method approach and did use some of the process data available in an attempt to explain the outcomes observed. We did not assess the use of global positioning devices (GPS) or information on distance from health facility as few evaluations used these devices, or had this information in their household surveys. GPS data, or data on distance from health facilities could be used to determine if CHWs are reaching the populations targeted (eg, hardest to reach areas) and can also be used in the analysis of inequities along with the wealth data.

Finally, we did not systematically review who was conducting the evaluation. For the most part, implementers collected process data and evaluators collected outcome data but sometimes evaluators collected process data and implementers collected outcome data. In addition, sometimes implementers conducted the entire evaluation, and sometimes evaluators conducted the entire evaluation. It is now recommended that stakeholders, implementers, and evaluators should work together, plan prospectively for the evaluation and implementation research to ensure all data elements suggested are collected, that program management is well documented, and data are periodically reviewed and used for program improvement [38,39]. Evaluation and implementation research is most likely to be more comprehensive, useful and result in actual change if stake holders and implementers are not just a passive recipient of results.

At the time of writing of this article, many of these evaluations are not yet complete and of those that are completed, several have not been published or are not available to the public. Evaluation results need to be widely disseminated with iCCM program implementers and supporting partners as soon as possible after completion, so that others may learn and benefit from these evaluations. We suggest that these reports be made available to the public through the donor websites and websites dedicated to community based treatments in the developing world. We also encourage all the researchers to publish these data as soon as feasible.

Regardless of the availability of reports, there has been an increase in the number of evaluations of iCCM in Africa completed in the past 5 years. This has been driven by donors, for the most part, requesting impact evaluations, although our review demonstrated that process and contextual information is critical to better implement programs in real world settings. We were able to use most of these evaluations to do a multi–country review of aspects of iCCM that are associated with higher utilization of iCCM, also presented in this supplement; however, if an emphasis had been instead on process and outcome evaluations or implementation research using standardized indicators we would have been able to use all these studies and done a more in–depth analysis [41]. More engagement is needed with funders regarding the appropriateness of conducting impact evaluations too early in implementation phase and without complete process, contextual and outcome data because the results are likely to be misleading to policy makers and will not reflect the true potential of these interventions. If donors and governments requesting evaluations of iCCM in the Africa region provide sufficient resources to conduct evaluations and inform evaluators to follow our suggested key data, elements and design for future evaluations, we should be able to pool more data in the future to better determine the impact of iCCM on child morbidity and mortality in the Africa region.
