# Supplementary material for: A proposed model to conduct process and outcome evaluations and implementation research of child health programs in Africa using integrated community case management as an example
Source: J Glob Health. 2014 Dec;4(2):020409. doi: 10.7189/jogh.04.020409 (PMC4267085; doi:10.7189/jogh.04.020409)
Supplement: Online Supplementary Document [file jogh-04-020409-s001.pdf]

Online Supplementary Document

Diaz et al. A proposed model to conduct process and outcome evaluations and implementation research of child health programs in Africa using integrated community case management as an example.
J Glob Health 2014;4:020409

Table 1. Detailed Study descriptions

Country (Study organization)	Objective of evaluation	Design	Study area	Timeframe of study
**Burkina Faso (**Special Programme for Research and Training in Tropical Diseases, WHO**,** Groupe de Recherche Action en Santé)	To evaluate the impact on all-cause mortality in children aged 6 to 59 months, achievable through a home and community approach for integrated management of fevers/malaria and pneumonia by adding an antibiotic to antimalarial treatment	Cluster-randomized stepped wedge (three arms).	2 of 63 health districts (with villages as clusters).First year three arms are the controls. Second year arm 1 is control, arms 2 (malaria tx) and 3 (malaria and pneumonia tx).Third year arms 1 and 2 malaria tx and arm 3 malaria and pneumonia tx.	June 2009 – 2012
**Cameroon (Population Services International**	What is the impact of community based treatment of malaria and diarrhea on mortality (CHWs did not treat pneumonia)	Quasi-experimental pre-test/post-test	Two districts in the East region of Cameroon: Doumé and Nguelemendoukats	Oct 2009-2013
**Ethiopia Oromia**(John Hopkins University)	What is the mortality impact of adding pneumonia treatment to diarrhea and malaria treatment provided by Health Extension Workers?	Cluster-randomized step-wedge with stratification by zone	16 intervention and 15 control woredas of 670 rural woredas (control zone had iCCM diarrhea + malaria)	February 2011 – early 2013
**Ghana (**Special Programme for Research and Training in Tropical Diseases, WHO**,** School of Public Health, University of Ghana)	To evaluate the impact on all-cause mortality of adding an antibiotic to antimalarial treatment of 2-59 months old children with fever within the Home Management of Malaria (HMM) strategy	Cluster-randomized stepped wedge	1 district of 17 districts in Greater Accra Region, group of communities served as clusters. First year three arms are the controls. Second year arm 1 is control, arms 2 (malaria tx) and 3 (malaria and pneumonia tx).Third year arm 2 and 3 remain the same, arm one half receive malaria half receive malaria and pneumonia	Jan 2006 – Dec 2009

			tx.	

iCCM = Intergrated Community Case Manangement, Tx = treatment, CHW = community health worker

Country (Study organization)	Objective of evaluation	Design	Study area	Timeframe of study
**Ghana** (South Africa Medical Research Council) on behalf of UNICEF)	To evaluate iCCM using the DAC Criteria for Evaluating Development Assistance	Pre-test/post-test no comparison area	28 of 28 districts in 3 regions (UE, UW and northern)	2012-2014
**Malawi** (Save the Children)	To evaluate the change in coverage when iCCM is implemented	Quasi-experimental step-wedge with comparison at midline but all areas with intervention at endline	1 of 29districts (targeting hard to reach areas)	2009-2013
**Malawi** (South Africa Medical Research Council on behalf of UNICEF)	To evaluate iCCM using the DAC Criteria for Evaluating Development Assistance	Pre-test/post-test no comparison area	10 of 29districts (targeting hard to reach areas)	2012-2014
**Mozambique** (Save the Children)	To evaluate the coverage outcome of iCCM	Quasi-experimental post-test only with comparison area	3 intervention and 1 comparison district of 148 districts	2009-2012
**Niger** (South Africa Medical Research Council on behalf of UNICEF)	To evaluate the iCCM using the DAC Criteria for Evaluating Development Assistance and documentation of implementation as part of Catalytic Initiative	Pre-test/post-test no comparison area	42 of 42 districts	2012-2014
**Rwanda** (International Rescue Committee)	To assess changes in treatment coverage for pneumonia, malaria and diarrhea	Pre-test/post-test no comparison area	6 of 30 districts	2007-2011
**Sierra Leone** (International Rescue Committee)	To evaluate the outcome and impact of implementing iCCM	Semi-randomized stepped wedge trial design.	4 of 12 districts	2006-2013

Country (Study Organization)	Objective of Evaluation	Design	Study Area	Timeframe of study
**Sierra Leone** (UNICEF)	To evaluate the outcome and impact of implementing iCCM	Quasi-experimental pre-test/post-test	2 implementing + 2 control districts of 12 districts	2009-2013
**South Sudan** (International Rescue Committee)	Impact evaluation of the iCCM program as implemented in Panijar County	Quasi-experimental pre-posttest intervention area, comparison area post-test only	1 intervention county + 1 control county of 86 counties	2005-2012
**South Sudan** (Malarial Consortium)	Impact evaluation of the iCCM program as implemented in xxx County	Quasi-experimental pre-posttest intervention area, comparison area post-test only	1 intervention county + 1 control county of 86 counties	2009-2012
**South Sudan** (Save the Children)	Impact evaluation of the iCCM program as implemented in Mvolo and Kapoeta counties	Quasi-experimental pre-posttest intervention area, comparison area post-test only	2 intervention counties + 1 control county of 86 counties	2009- June 2012
**Uganda Central** (Malaria Consortium andUNICEF)	To evaluate the outcome and impact of implementing iCCM	Quasi-experimental pre-test/post-test	3 intervention and 3 control districts	2009-2012
**Uganda East (**Special Programme for Research and Training in Tropical Diseases, WHO**,** Makerere University)	To determine the impact on under 5 mortality achievable through an integrated approach for home and community presumptive treatment of malaria and pneumonia in children 4 – 59monthsby adding an antibiotic to antimalarial treatment	Cluster-randomized trial	Iganga-Mayuge Health and Demographic Surveillance Site, villages served as clusters	2009-2012
**Uganda West** (Malaria Consortium)	To evaluate the outcome and impact of implementing iCCM	Quasi-experimental pre-test/post-test	8 intervention and 3 control districts	2010-2013
**Zambia** (MalariaConsortium)	To evaluate the outcome and impact of implementing iCCM	Quasi-experimental, comparison at post test	4 intervention and 3 control districts (phased-in)	2010-2012

Country (Study organization)	Objective of evaluation	Design	Study Area	Timeframe of study
**Ethiopia** (South Africa Medical Research Council on behalf of UNICEF)	To evaluate the iCCM using the DAC Criteria for Evaluating Development Assistance and documentation of implementation as part of Catalytic Initiative	Pre-test/post-test no comparison area	Full support by UNICEF in 239 woredas in 26 zones in the Tigray, Amhara, SNNP, Oromia and Benishangul-Gumuz regions.Partial support in an additional 32 zones and 340 woredas of Oromia, Tigray, Amhara, SNNP, Afar and Gambella.	2012-2014
**Mali**(South Africa Medical Research Council on behalf of UNICEF)	To evaluate the iCCM using the DAC Criteria for Evaluating Development Assistance	Pre-test/post-test no comparison area	5 southern regions	2012-2014
**Mozambique** (South Africa Medical Research Council on behalf of UNICEF)	To evaluate the iCCM using the DAC Criteria for Evaluating Development Assistance	Pre-test/post-test no comparison area	national	2012-2014
**Mozambique** (Malaria Consortium)	To evaluate the change in coverage when iCCM is implemented	Pre-test/post-test no comparison area	7 districts (another 6 with UNICEF)	2010-2012
**Zambia** (Boston University)	To assess the effectiveness of CHWs managing malaria and pneumonia	Cluster-randomized controlled trial	15 intervention community health post (CHP) catchment areas and 16 control CHP catchment areas in Chikankata Mission Hospital catchment area covering two districts in Southern Province, Zambia	2007-2008
